# Preparation and structures of PEBA gas separation membrane modified by fumed silica for oil vapor separation

**DOI:** 10.1038/s41598-022-05064-7

**Published:** 2022-01-19

**Authors:** Rong Xu, Beifu Wang, Yuting Cai

**Affiliations:** 1 School of Naval Architecture and Maritime, Zhejiang Ocean University, Zhoushan, 316000 Zhejiang China; 2 School of Petrochemical Engineering and Environment, Zhejiang Ocean University, Zhoushan, 316000 Zhejiang China

**Keywords:** Environmental sciences, Materials chemistry, Atmospheric chemistry

## Abstract

Composite membranes were fabricated with polyethersulfone as a microporous substrate and polyether block amide (PEBA) as a selective layer to achieve efficient recovery of volatile organic compounds (VOCs). Fumed silica was mixed into PEBA for modification. The top thin layers with different percentage of fumed silica in PEBA were prepared by spin-coating. Structure and performance of membranes with and without a modification were characterized. The results showed that fumed silica in an ultra-thin selective layer significantly influenced the hydrophobicity of the membranes. The higher the content of fumed silica, the higher the hydrophobicity of the membranes was. The maximum content of added fumed silica was 0.6 wt%. When the proportion of fumed silica reached 0.6 wt%, the contact angle could reach 95.8°, which was 56% higher than that of the unmodified one. The structure of the membrane remained unchanged. Moreover, the separation performance was evaluated by removing VOCs from a mixture of oil vapor and nitrogen. The VOCs permeance tended to grow with an increase in the content of fumed silica. When the content was 0.6 wt%, the membrane exhibited better comprehensive performance. Its vapor flux rate was 117.8 ml/min, which was 153% higher than that without a modification. Its separation coefficients for ethane, propane, cyclopropane, isobutane and n-butane were 29.3, 29.9, 24.9, 30.7, and 34.0 respectively.

## Introduction

In recent years, air pollution has attracted great attention, especially volatile organic compound (VOC) pollution. Under the action of sunlight, VOCs react with NO_x_ and produce secondary aerosol, which is an important precursor of air pollution. Therefore, controlling the emission of VOCs can reduce the concentration of PM2.5, relieving air pollution^1,2^. In addition, VOCs in the atmosphere can react further with ozone, leading to the destruction of the ozonosphere^3,4^. Oil vapor, being an important air pollutant, is a VOC pollutant. With an increase in crude oil consumption, a large amount of oil vapor has been produced during the storage and transportation of crude oil. Oil vapor has a complex composition, high pollution concentration, and long duration, which has a significant impact on air quality. Long-term exposure to VOCs can cause irreversible permanent harm to the human body^5^. In order to protect the environment, universities and research institutes actively investigate relevant topics to prevent and control pollution^6^.

Traditionally, oil vapor is oxidized and decomposed into non-toxic or low-toxic substances through chemical and biochemical reactions. Physical methods such as adsorption, absorption, and condensation can also be adopted, but the cost is high and the economic benefit is low^7–9^. Compared with traditional treatment methods, the membrane method has the advantages of low cost, high efficiency, low energy consumption, simple operation, and no secondary pollution. Hence, its in-depth study is required^10–12^.

Polyether block amide (PEBA) is a typical commercial rubber block copolymer with high thermal stability, mechanical stability, acid–base tolerance, and organic solvent resistance, which has become promising material. However, its gas flux and separation effect are poor for specific compounds. Reijerkerk et al. prepared a PEBA1657 composite membrane with 80% polyethylene glycol (PEG) and 20% polydimethylsiloxane as additives, and studied its gas separation performance^13^. Dong et al. added a supramolecular polymer (F127-Tpy-M) to PEBA2533 to prepare a composite membrane for gas separation^14^. Therefore, a modification is necessary for PEBA membranes to improve their gas flux and separation performance.

Robeson has proposed that there is a balance between permeability and permselectivity in polymer membranes. This balance is called the Robeson upper bound^15^. Its trade-off effect is that an increase in permeability is at the expense of permselectivity. The separation behaviour of inorganic membranes exceeds the Robeson bound. Therefore, combining above two materials to prepare composite membranes has become a trend. Research has shown that high-silica zeolite, an ideal adsorbent for VOCs, exhibits good hydrophobicity and stability. Thus, hydrophobic fumed silica was selected as an inorganic material in this study. Fumed silica is nano-silica prepared by the gas-phase method. The surface of hydrophobic fumed silica is covered with non-polar methyl groups. Because of its hydrophobicity and VOC absorption characteristics, fumed silica and PEBA were mixed to prepare a gas separation membrane in order to enhance gas separation performance.

In this study, polyethersulfone (PES) was used as a material of the supporting layer and a PEBA selective layer was prepared by spin-coating. Fumed silica was applied to modify the PEBA separation layer. The effect of the addition of fumed silica on the structure and properties of the membrane was discussed. The membranes were characterized by scanning electron microscope (SEM), attenuated total internal reflectance Fourier transform infrared spectroscopy (ATR-FTIR), X-ray photoelectron spectroscopy (XPS), and contact angle, gas flux and separation tests.

## Materials and methods

### Materials

PES was purchased from Solvay S.A. (USA). Chemicals including N-methylpyrrolidone (NMP) (AR, > 98%), polyvinyl pyrrolidone (PVP), n-butyl alcohol (AR, > 99.7%), and PEBA were obtained from Sinopharm Chemical Reagent Co., Ltd. (Shanghai, China). Fumed silica was supplied from Wacker Chemie AG (Germany).

A mixture of liquefied petroleum gas and nitrogen was used as simulated gas. Liquefied petroleum gas (LPG) was purchased from Zhoushan LPG Company.

### Preparation of a PES membrane

PES and PVP were dried in an oven at 60 °C for 24 h to a constant weight. The proportion of PES, NMP, and PVP was 18:72:10 by weight. They were mechanically stirred at 90 °C for 24 h until a uniform and stable casting solution was obtained. The casting solution was placed in a 90 °C oven for 24 h to remove bubbles. Next, the casting solution was poured onto a dry and smooth glass plate (14.5 cm × 30 cm). The membrane was casted with a medical knife and then placed into deionized water. Subsequently, deionized water was changed, and the membrane was left for 24 h to release the solvent after its complete stripping from the glass plate.

### Preparation of a PEBA separation membrane

PEBA and fumed silica were dried in an oven at 60 °C for 24 h to a constant weight. Certain amounts of PEBA, fumed silica, and n-butanol were mixed and placed in an Erlenmeyer flask. The bottle mouth was sealed with a polyethylene (PE) film to prevent n-butanol from volatilizing. The Erlenmeyer flask was placed in a 90 °C water bath, and the casting solution was stirred for 4 h. The casting solution was ultrasonically treated to ensure uniform dispersion of fumed silica. Finally, a 7 wt% PEBA casting solution was obtained. The casting solution was placed in a 90 °C oven for 12 h to remove bubbles.

The separation membrane preparation methods mainly include dip-coating, spin-coating and spray coating. The dip-coating method is suitable for low-concentration casting solutions. High viscosity of the casting solution can result in an uneven thickness of the separation membrane. The disadvantage of spray coating is that the nozzle is easily blocked when the casting solution concentration is high; thus, frequent replacement required. Spin coating is a traditional method for preparing separation membranes, which has the advantages of high cost effectiveness, energy saving, and controllable thickness accuracy. Membranes prepared by spin-coating have a uniform thickness, which is precisely controllable between 30 nm and 60 μm. Such membranes have a wide range of applications in the fields of biology, medicine, and other functional membranes^16–18^.

A separation membrane with micro-nano structures was prepared using the spin-coating method^19,20^. A PES membrane was cut into a 10 cm in diameter disc, and then, it was fully attached to the substrate. The casting solution of the separation membrane was coated on the PES membrane to prepare a composite membrane by the spin-coating method. Dynamic dripping was carried out at 500 rpm. After the low-speed rotation was completed, a high-speed rotation was performed at 2000 rpm so that the casting solution completely and uniformly covered the supporting membrane. The composite membranes were dried in a 60 °C oven for 12 h after spin coating.

A series of fumed silica modified PEBA membranes were prepared to investigate the effects of fumed silica on the structure, performance, gas flux, and separation effect of PEBA membranes. The content of fumed silica in the modified PEBA membranes was 0.0 wt%, 0.2 wt%, 0.4 wt%, 0.6 wt% and 0.8 wt%, and the corresponding composite membranes were labelled as PES/PEBA_0_, PES/PEBA_0.2_, PES/PEBA_0.4_, PES/PEBA_0.6_, and PES/PEBA_0.8_ respectively.

### Membrane characterization

#### SEM analysis

The composite membranes were observed by a Quanta200 scanning electron microscope (SEM) produced by Philip. Three samples were cut from each membrane, washed with distilled water, and dried as scanning samples. Samples were sprayed with gold, and the positive and negative sides were noticed during the treatment. Samples were collected by tweezers and scanned by SEM. The effect of the addition of fumed silica on the membrane structure was observed by SEM, and the maximum addition of fumed silica could be inferred.

#### ATR-FTIR analysis

Through the ATR-FTIR test, the characteristic peak of the sample and its corresponding functional groups were determined simply and quickly so as to speculate the structure of the sample and whether the chemical reaction was successful. The supporting membrane and the composite membrane before and after the modification were tested by a GJD2014125236-02 Fourier spectrometer (FT-IR6100) produced by Jasco Co., Japan. The wavenumber range was 500–4000 cm^−1^, the resolution was 0.07 cm^−1^, and the signal-to-noise ratio was 50,000:1.

#### XPS analysis

XPS was used to analyse the composition and valence of elements (except for H and He) on the surface of the separation membranes. In this experiment, K-alpha XPS was used to determine the change of the element content on the surface of separation membranes. The test depth was approximately 8 nm.

#### Contact angle test

In this experiment, the contact angle was referred to the angle of the water drop on the interface tangent to the sample membrane, which is a measure of the degree of wetting and a test of the hydrophilicity. Hydrophobic separation membranes are widely used in gas separation owing to their special hydrophobicity and selective separation characteristics. How to endow a separation membrane with excellent hydrophobicity had always been one of the main directions of gas separation membrane research^21^.

The wetting process was related to the interfacial tension of the system. When a drop of liquid falls on the surface of a horizontal membrane and reaches equilibrium, the formed contact angle and the interfacial tension conform to the following Eq. (1)^22^:

1 $$Y_{{{\text{SV}}}} = { }Y_{SL} + { }Y_{VL} {\text{cos}}\uptheta$$

where $Y_{{{\text{SV}}}}$, $Y_{SL}$ and $Y_{VL}$ represent the interfacial energy of solid–gas, solid–liquid, and gas–liquid interface respectively. θ is the angle between the gas–liquid interface and the solid–liquid interface. If θ < 90°, the membrane surface is hydrophilic; if θ > 90°, the membrane surface is hydrophobic. The effect of the addition of fumed silica on the hydrophilicity of the membrane can be analysed by the contact angle test.

#### Gas flux test

**Figure 1:**
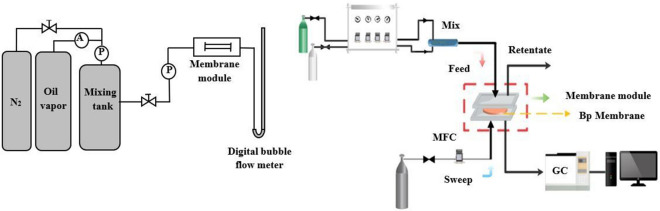
Device of the oil vapor separation test.

An oil vapor separation device was designed, as shown in Fig. 1a. Oil vapor and nitrogen were mixed in a tank. LPG and gasoline vapor were simulated VOCs. The pressure of the mixing tank was kept at 0.4 MPa. Another side of the membrane module remained at ambient pressure. The gas flux could be directly measured using a digital soap bubble flowmeter. The flux of the modified PEBA membranes with different amount of fumed silica were gauged to analyse the effect of fumed silica on the flux.

#### Separation performance test

According to the basic working principle of gas chromatograph (GC), the sample components could be quickly and qualitatively detected. The oil vapor before and after separation in the gas separation experiment was characterized and analysed by GC. The column used in the GC was KB-Al_2_O_3_/Na_2_SO_4_ (inner diameter: 0.53 mm and length: 50 m). The gas separation coefficient was used to characterize the rate difference of different gas components passing through the membrane so as to describe the gas separation performance of the membranes. It represents the ratio of the relative content of a component in the stream before and after the membrane separation. The separation efficiency of modified membranes with different fumed silica contents were compared and analysed. The device is depicted in Fig. 1b.

The separation coefficient of the membranes can be calculated by the following equations.

2 $$P = \frac{Ql}{{At\Delta p}}$$

3 $$\alpha_{A/B} = \frac{{P_{A} }}{{P_{B} }}$$

Herein, *P* is the gas permeability coefficient, barrer; *Q* is the volume of gas passing through the membrane at a certain time, cm^3^ (STP)/s; *t* is the test time, s; *A* is the effective area of the membrane, cm^2^; $\Delta p$ is the difference in pressure on both sides of the membrane, cmHg; *l* is the membrane thickness, cm; $P_{A} \left( {P_{B} } \right)$ is the gas permeability coefficient of gas A and B, barrer; $\alpha_{A/B}$ is the separation coefficient.

## Results and discussion

### SEM, ATR-FTIR, and XPS analysis results

**Figure 2:**
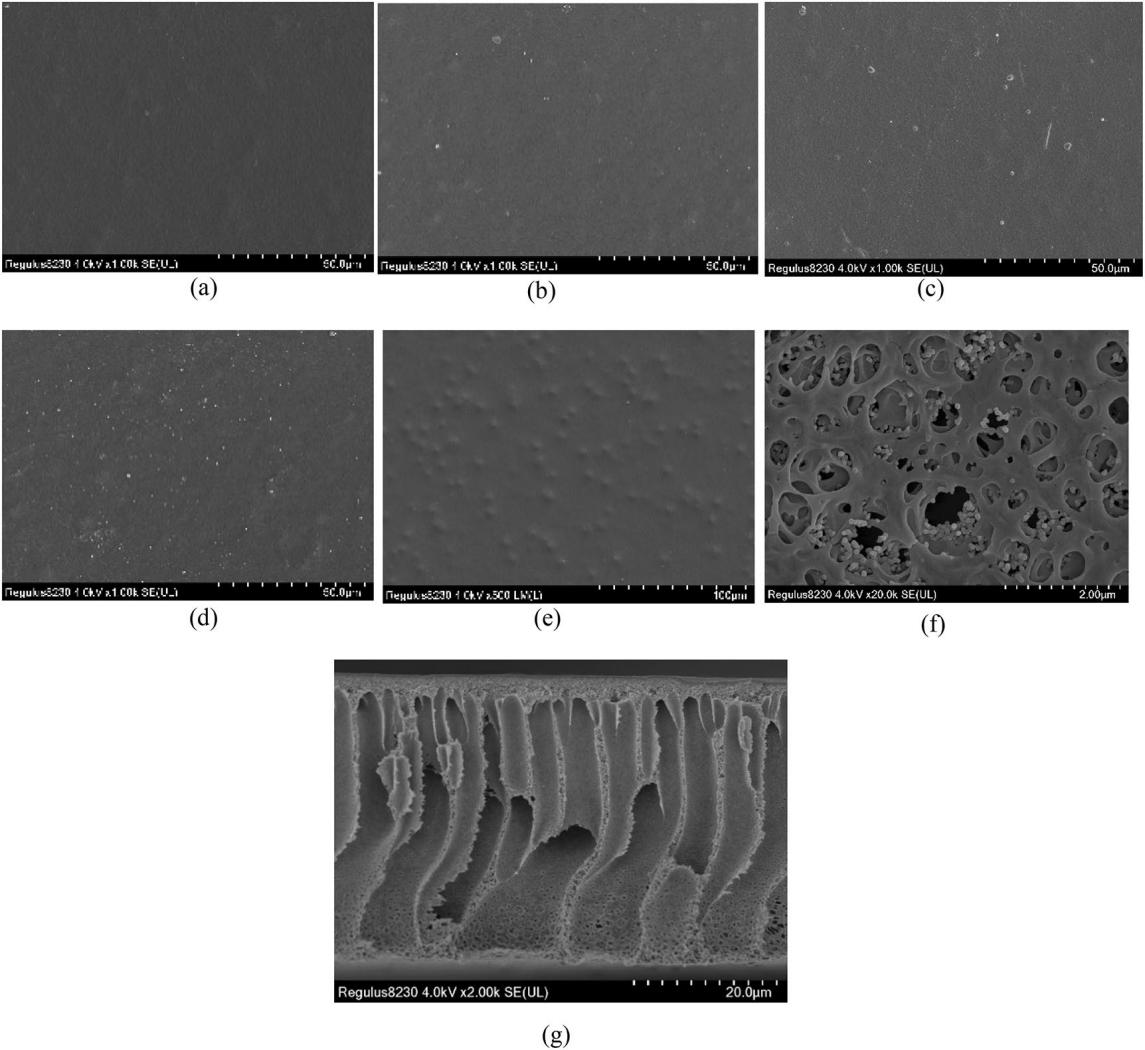
SEM images of composite membranes. (**a**–**e**) Front images of composite membranes, fumed silica contents are (**a**) 0.0 wt%, (**b**) 0.2 wt%, (**c**) 0.4 wt%, (**d**) 0.6 wt%, and (**e**) 0.8 wt%. (**f**): Reverse side image of PES/PEBA_0.6_. (**g**): Cross-section image of PES/PEBA_0.6_.

In Fig. 2, SEM images of composite membranes are presented. The front image is the surface of the selective layer, and the reverse side image is that of the pure supporting layer. From Fig. 2d,f,g, the supporting membrane appears to be porous, and the composite membrane surface becomes nonporous and dense after coating the separation membrane. Thus, it can be inferred that the separation membrane completely covers the supporting membrane.

It can be seen from Fig. 2a that the PEBA membrane has a uniform surface, a compact structure, good continuity, and no pores. When the content of fumed silica is 0.2 wt% (Fig. 2b) or 0.4% (Fig. 2c), the membrane surface is still smooth. When the content of fumed silica is 0.6 wt% (Fig. 2d), micro-agglomeration is observed, but fumed silica is still well dispersed in the membrane. When the content of fumed silica is 0.8 wt% (Fig. 2e), owing to the force between the particles, fumed silica begins to aggregate in the form of block particles. Large aggregates and bulges are observed on the membrane surface, indicating that fumed silica in the membrane has been excessive and cannot diffuse uniformly.

**Figure 3:**
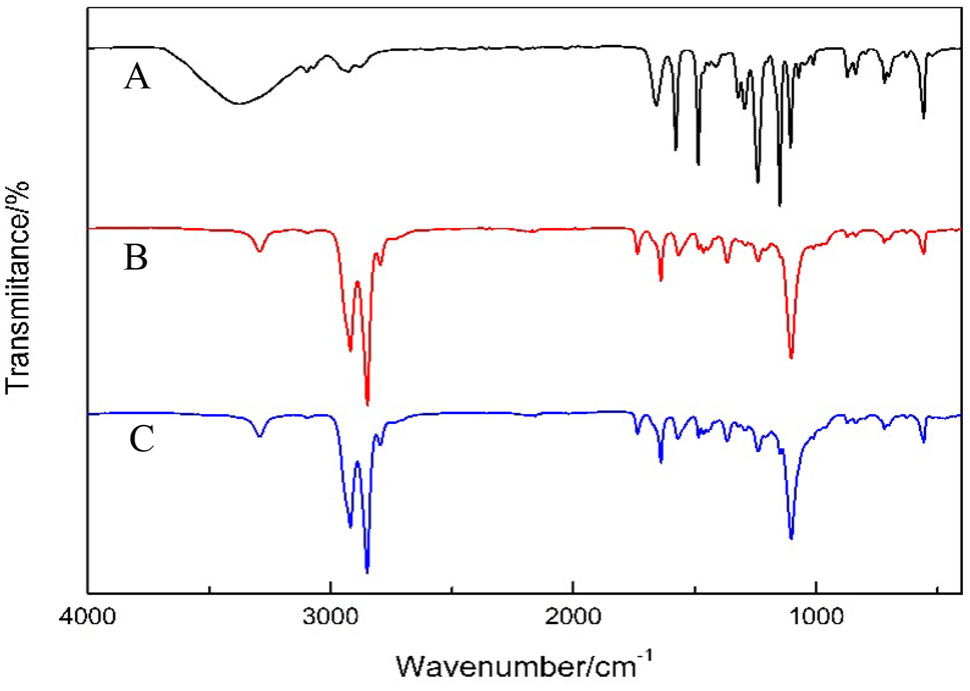
FTIR spectra A (supporting membrane), B (composite membrane) and C (modified membrane).

Figure 3 shows ATR-FTIR spectra of the PES supporting membrane, PEBA separation membrane, and PEBA separation membrane modified by fumed silica. The absorption peaks of PES disappear, and the absorption peaks of PEBA appear in spectra A and B. Together with SEM analysis results, the ATR-FTIR results show that the PEBA separation membrane completely covers the PES supporting membrane.

FTIR spectra B and C absorption peaks at 1100 cm^−1^ and 1240 cm^−1^ corresponding to C–O–C group, 1567 cm^−1^ and 1640 cm^−1^ corresponding to O=CNH group on PA, 1731 cm^−1^ corresponding to C=O group, 2850 cm^−1^ and 2924 cm^−1^ corresponding to C-H group, and 3287 cm^−1^ corresponding to N–H characteristic peak. The positions of each peak are consistent with the results presented in the literature^23^.

The position of the basic characteristic peak of PEBA does not change. The molecular structure of the membrane is unchanged before and after the modification. Therefore, the addition of fumed silica does not affect the chemical structure of the PEBA separation membranes.

**Figure 4:**
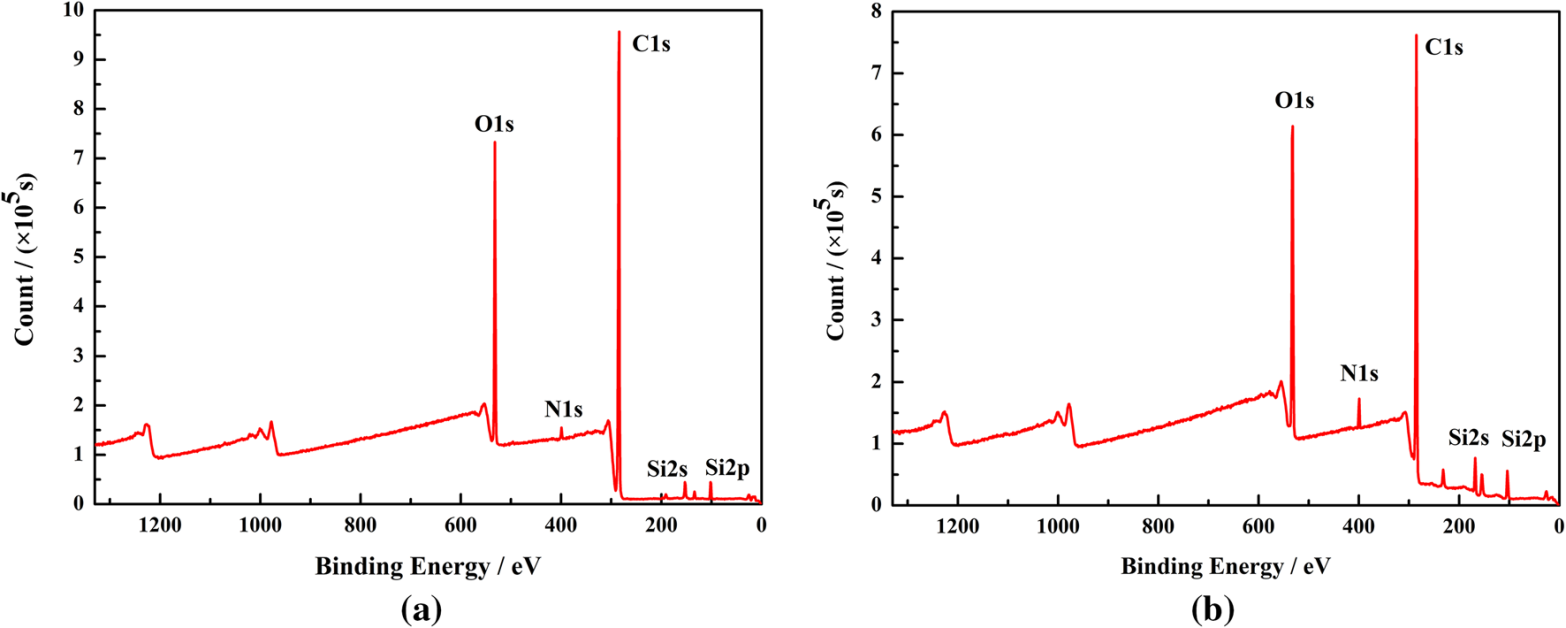
The XPS survey spectra of (**a**) PES/PEBA_0_ and (**b**) PES/PEBA_0.6_ separation membrane surfaces.

XPS was used for further analysing the effect of the addition of fumed silica on the chemical structure of the PEBA separation membranes. Figure 4 demonstrates XPS survey spectra of PES/PEBA_0_ and PES/PEBA_0.6_ separation membrane surfaces. In Fig. 4, spectral peaks of C, O, N, and Si are visible in the separation membranes. The binding energy peak at 103.5 eV is attributed to Si in fumed silica. From comparison of Fig. 4a,b, the contents of Si and O atoms increase by 2.17% and 5.54%, respectively. The content of other atoms decreases, and the position of the spectral peak remains unchanged.

In conclusion, the XPS results are basically consistent with the ATR-FTIR analysis results. Therefore, it can be concluded that the addition of fumed silica does not affect the chemical structure of the PEBA separation membranes. The large specific surface area and hydrophobicity of fumed silica enhance the hydrophobicity of the separation membranes, which is conducive to the separation of VOCs.

### Influence of fumed silica content in casting solution on the membrane’s hydrophobicity

**Figure 5:**
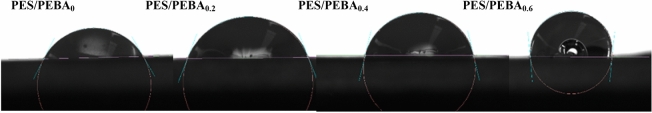
Contact angle of modified PEBA membranes with different fumed silica contents.

Figure 5 shows the contact angles on the surface of PEBA modified membranes with different fumed silica contents. The contact angle of PES/PEBA_0_ is 61.5°, which is consistent with the property that PEBA is a hydrophilic polymer. The contact angles of PES/PEBA_0_, PES/PEBA_0.2_, PES/PEBA_0.4_, and PES/PEBA_0.6_ correspond to 61.2°, 68.2°, 76.3°, and 95.8°, respectively.

The contact angle and the hydrophobicity increase with the fumed silica content. Combined with the properties of fumed silica, its surface is connected with non-hydrolytic groups such as methyl. Hydrophobic fumed silica was added into the separation membrane for blending modification, which enhanced the hydrophobicity of the separation membrane. It can be seen that fumed silica cannot be uniformly dispersed from SEM photo and begins to aggregate in the form of block particles. Therefore, the hydrophobicity reaches the maximum when the amount of added fumed silica is 0.6 wt%.

### Influence of fumed silica content in casting solution on the membrane’s gas flux

**Figure 6:**
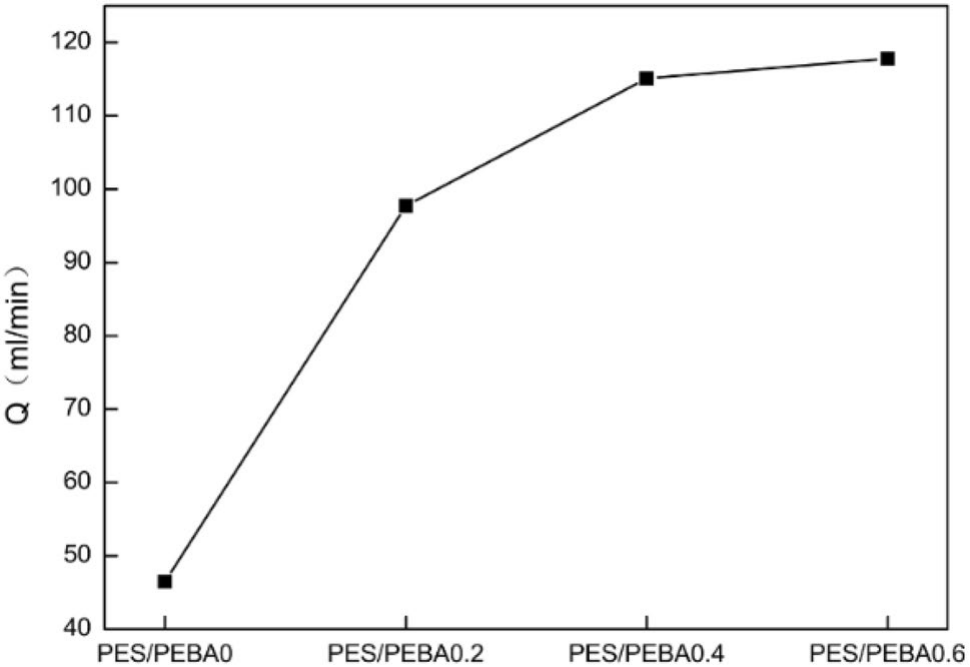
Gas flow rate of modified PEBA membranes with different fumed silica contents.

It can be seen from Fig. 6 that the gas volume flux of the composite membranes increases with the fumed silica content. The gas volume flows of PES/PEBA_0_, PES/PEBA_0.2_, PES/PEBA_0.4_, and PES/PEBA_0.6_ are 46.5, 97.7, 115.1, and 117.8 ml/min, respectively.

With the addition of fumed silica, the flux increases significantly mainly because fumed silica affects the diffusion separation process of gas in the PEBA membranes. There are two main diffusion paths in the process of gas separation: Knudsen diffusion mechanism and dissolution diffusion mechanism. The Knudsen diffusion mechanism usually exists in the transfer process of gas through the molecular pores in the filler particles. This phenomenon can also be found when there is an interface gap between the filler particles and the polymer matrix^24^. Gas transport through polymers is usually carried out via the dissolution diffusion mechanism, which is related to the ability of gas molecules to penetrate the polymer surface membrane. The dissolution diffusion mechanism can be realized by promoting the transport of permeation gas. The functional groups attached to the fumed silica particles act as mobile carriers to transport the expected permeation gas through the polymer of the membrane^25^.

With the addition of fumed silica, the nonselective void of the membrane increases. The Knudsen diffusion mechanism dominates, and the gas enters through the nonselective void between the polymer and the fumed silica particles. The diffusion path provided by the nonselective gap between PEBA and fumed silica increases the gas flux. The higher the content of fumed silica, the more non-selective voids provide the diffusion path, and the flux increases gradually.

### Effect of fumed silica content in casting solution on the separation performance of membrane

#### LPG separation test

**Figure 7:**
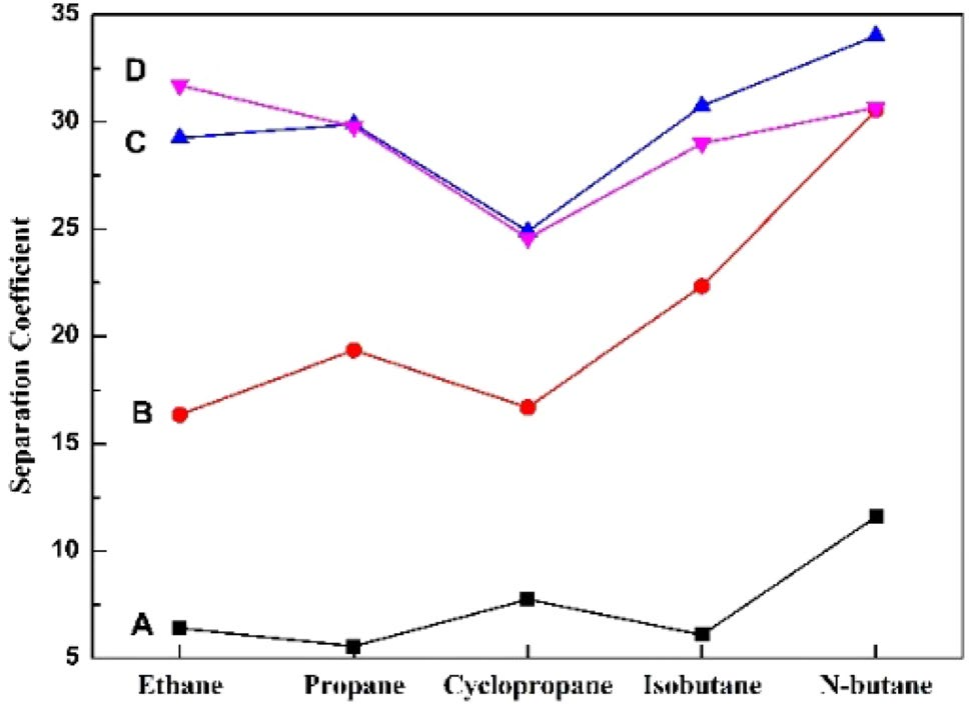
LPG separation performance of modified PEBA membranes with different fumed silica contents. (Lines A, B, C, and D correspond to PES/PEBA_0_, PES/PEBA_0.2_, PES/PEBA_0.6_ and PES/PEBA_0.4_, respectively).

The dominant components in oil vapor are similar to the main components of liquefied petroleum gas. Liquefied petroleum gas was used as a simulated gas for the separation test. According to the gas chromatographic analysis and calculation, the gas separation coefficients of modified PEBA membranes with different fumed silica contents were obtained. The separation ability of the membranes was analyzed by investigating the separation coefficients of different components. The separation coefficients of different hydrocarbons are shown in Table 1. The separation performance of modified PEBA membranes with different fumed silica contents for each component is shown in Fig. 7.

**Table 1:** The separation coefficients of different gases tested by membranes with different fumed silica contents.

	PES/PEBA_0_	PES/PEBA_0.2_	PES/PEBA_0.4_	PES/PEBA_0.6_
Ethane	6.4	16.4	31.7	29.3
Propane	5.6	19.4	29.8	29.9
Cyclopropane	7.7	16.7	24.6	24.9
Isobutane	6.1	22.3	29.0	30.7
N-butane	11.6	30.6	30.7	34.0

It can be seen from Fig. 7 that fumed silica improves the separation performance of the membranes for each component. Compared with PES/PEBA_0_, PES/PEBA_0.2_ improves the separation effect of n-butane most significantly, and the separation coefficient increases from 11.6 to 30.6, which is 2.6 times higher than that without a fumed silica membrane. PES/PEBA_0.4_ enhances the separation performance of ethane and propane most obviously. The separation coefficient of ethane increases from 6.4 to 31.7, and that of propane increases from 5.6 to 29.8, which are 5.0 times and 5.3 times higher than those without a fumed silica membrane, respectively.

The separation performance of PES/PEBA_0.4_ and PES/PEBA_0.6_ for each component is similar, but PES/PEBA_0.6_ better separates isobutane and n-butane. When the content of fumed silica is 0.6 wt%, the separation performance reaches the maximum. Its separation coefficients of ethane, propane, cyclopropane, isobutane, and n-butane are 29.3, 29.9, 24.9, 30.7, and 34.0, respectively.

Afnan et al. prepared a block co-polyimide 6FDA-CARDO/6FDA-Durene (2500/2500) membrane, Its comprehensive separation coefficient was 28. At 36 vol% H_2_S, the H_2_S/CH_4_ separation coefficient was 24^26^. Yang and coworkers doped inorganic filler particles of a 4A molecular sieve and a NaY molecular sieve into a PEBA matrix to prepare mixed matrix by the blending method. The experimental results showed that the separation coefficients of ethylene and propylene mixture with a ratio of 1:1 were 5.15 and 8.84, respectively. Yang et al. tested the separation performance of a PDMS/ceramic composite membrane; the VOC/N_2_ separation coefficient was 27.1^27^. In the present experiment, the gas separation performance of the PES/PEBA membrane modified by fumed silica is not lower than that of the membranes above. This remarkable membrane separation performance shows that the membrane has a good potential application prospect in VOC separation.

#### Gasoline vapor separation test

**Figure 8:**
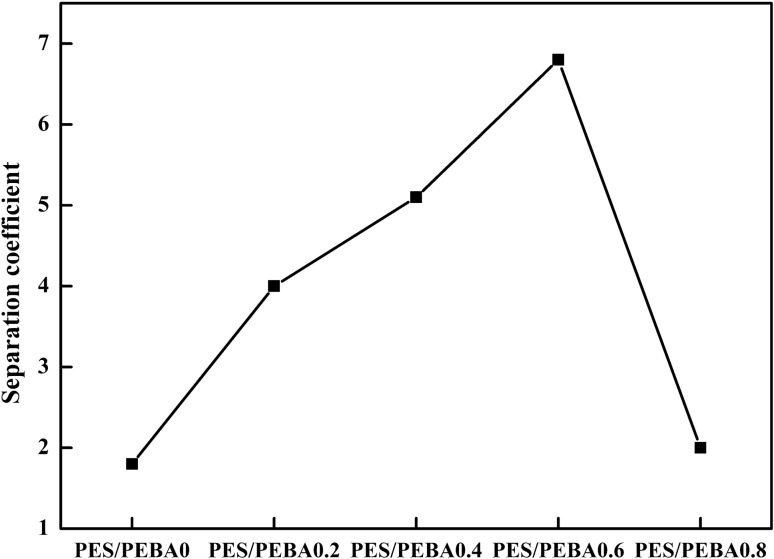
Gasoline vapor separation coefficient of modified PEBA membranes with different fumed silica contents.

Gasoline vapor was used for separation to test the separation performance of the composite membrane. The gasoline was bubbled with nitrogen to collect gasoline vapor, and a separation device was used. From comparison of the separation effects of the membrane on gasoline vapor and LPG, it was verified that the addition of fumed silica could improve the membrane performance to separate organic gas. The separation coefficients of modified PEBA membranes with different fumed silica contents are showed in Fig. 8.

It can be seen from Fig. 8 that when the content of fumed silica is 0.8 wt%, the separation performance decreases sharply because fumed silica has been excessive and cannot diffuse evenly, resulting in the reduction in the separation capacity.

The performance to separate gasoline vapor is directly proportional to the content of fumed silica before fumed silica reaches the saturation concentration. When the content of fumed silica is 0.6 wt%, the separation coefficient is the largest (6.8). This result is consistent with the test of LPG separation.

The addition of fumed silica can change the gas separation performance of a membrane^28^. Ahn et al.^29^ explains that the existence of void volume at the interface between nanoparticles and polymers is the main factor for the enhancement of gas permeability of the modified membrane. Shen and Lua^30^ believe that the explanation of this phenomenon is related to the free volume theory, that is, nonporous silica can destroy the accumulation of polymer chains and increase the free volume of polymers. Fumed silica provides a suitable position for gas adsorption at the interface of the PEBA polymer chain, resulting in an increase in gas permeability. Under the action of low—concentration fumed silica, the solution diffusion mechanism is dominant because the nonselective gap between silica and polymer does not provide a sufficient diffusion path, resulting in an increase in the membrane selectivity.

## Conclusions

In this study, gas separation membranes were prepared using PES as the supporting membrane and PEBA as the separation membrane. Fumed silica was mixed into the separation membrane for modification. Modified membranes with different contents of fumed silica were prepared by the spin-coating method. Next, the membranes were characterized by SEM, ATR-FTIR, XPS, contact angle test and separation performance test. The results show that the maximum content of added fumed silica is 0.6 wt%. At the same time, the hydrophobicity of the membranes decreases greatly. When the content of fumed silica is 0.6 wt%, the maximum water contact angle is 95.8°, which is 56% higher than that of the membrane without fumed silica. It is also found the gas flow rate and the separation performance positively correlates with the change in the fumed silica content. When the content of fumed silica is 0.6 wt%, the gas flow rate is 153% higher than that of the membrane without fumed silica. Furthermore, the separation performance of n-butane and isobutane is increased by 193% and 405%, respectively.
