# Author Correction: Preparation and structures of PEBA gas separation membrane modified by fumed silica for oil vapor separation

**DOI:** 10.1038/s41598-022-07732-0

**Published:** 2022-03-01

**Authors:** Rong Xu, Beifu Wang, Yuting Cai

**Affiliations:** 1 School of Naval Architecture and Maritime, Zhejiang Ocean University, Zhoushan, 316000 Zhejiang China; 2 School of Petrochemical Engineering and Environment, Zhejiang Ocean University, Zhoushan, 316000 Zhejiang China

Correction to: *Scientific Reports* https://doi.org/10.1038/s41598-022-05064-7, published online 19 January 2022

In the original version of this Article, Rong Xu was incorrectly listed as a corresponding author. The correct corresponding author for this Article is Beifu Wang. Correspondence and request for materials should be addressed to wangbeifu@126.com.

The original Article has been corrected.

